# Carrier dynamics of Mn-induced states in GaN thin films

**DOI:** 10.1038/s41598-017-06316-7

**Published:** 2017-07-19

**Authors:** Yu-Ting Chen, Chi-Yuan Yang, Po-Cheng Chen, Jinn-Kong Sheu, Kung-Hsuan Lin

**Affiliations:** 1 Institute of Physics, Academia Sinica, Taipei, 11529 Taiwan; 2 Department of Physics, National Taiwan University, Taipei, 10617 Taiwan; 3 Department of Photonics, National Cheng Kung University, Tainan, 70101 Taiwan; 4 Institute of Photonics Technologies, National Tsing Hua University, Hsinchu, 30013 Taiwan

## Abstract

GaN-based materials are widely used for light emission devices, but the intrinsic property of wide bandgap makes it improper for photovoltaic applications. Recently, manganese was doped into GaN for absorption of visible light, and the conversion efficiency of GaN-based solar cells has been greatly improved. We conducted transient optical measurements to study the carrier dynamics of Mn-doped GaN. The lifetime of carriers in the Mn-related intermediate bands (at 1.5 eV above the valence band edge) is around 1.7 ns. The carrier relaxation within the Mn-induced bandtail states was on the order of a few hundred picoseconds. The relaxation times of different states are important parameters for optimization of conversion efficiency for intermediate-band solar cells.

## Introduction

GaN is pivotal for light emission devices^1–4^ and THz acoustic spectroscopy^5–7^, but its intrinsic property of wide bandgap (around 3.4 eV) makes it improper for photovoltaic applications. Conversion efficiency is a key parameter for solar cells, and there is a tradeoff between the bandgap and the solar spectrum^8^. Although low-bandgap material absorbs more solar frequencies, most photoexcited electrons gain too much extra energy, leading to waste heat. According to the Shockley and Queisser model, the maximum efficiency is ~ 40% for p-n junction solar cells based on single semiconductor^8^. Tandem cells (based on III-IV semiconductors) have been experimentally demonstrated to perform best (over 40%)^9^ up to now, but the fabrication cost is high. Luque and Marti proposed that the conversion efficiency of solar cells could increase by intermediate band (IB) within the bandgap of the material^10^. According to their model, the theoretical efficiency could be up to 63%, exceeding that of ideal two-terminal tandem cells which use two semiconductors^10^. Recently, significant increasing of experimental efforts^11–14^ have been devoted to improving the conversion efficiency of solar cells by using IBs in materials such as quantum dots^15–18^ and chalcopyrites^19–21^. Manganese was also doped into GaN to induce extra states for absorption of visible light, and the conversion efficiency of solar cells based on Mn-doped GaN was greatly improved^22–25^. Compared with tandem cell structure, the practical conversion efficiency for single material with IB is still low. The practical challenges, such as the impurity states with nonradiative relaxation, still remain for single material with IB.

Despite there is literature of carrier dynamics in GaN with different doping types^26–32^, lifetimes of carriers in Mn-doped GaN is unknown for applications of photovoltaics. The time-resolved information such as carrier lifetimes in each state are important for modeling^13^, because the absorption of photons within the forbidden gap is via the IBs as the step to generate free carriers. We conducted transient optical measurements to study the carrier dynamics of Mn-doped GaN. The lifetime of carriers in the Mn-related IB (at 1.5 eV above the valence band edge) is around 1.7 ns. Carrier relaxation within the bandtail states, below the conduction band edge, is on the order of a few hundred picoseconds. Understanding the carrier dynamics should lead to a way to improve the efficiency of practical devices.

## Experimental Design

**Figure 1:**
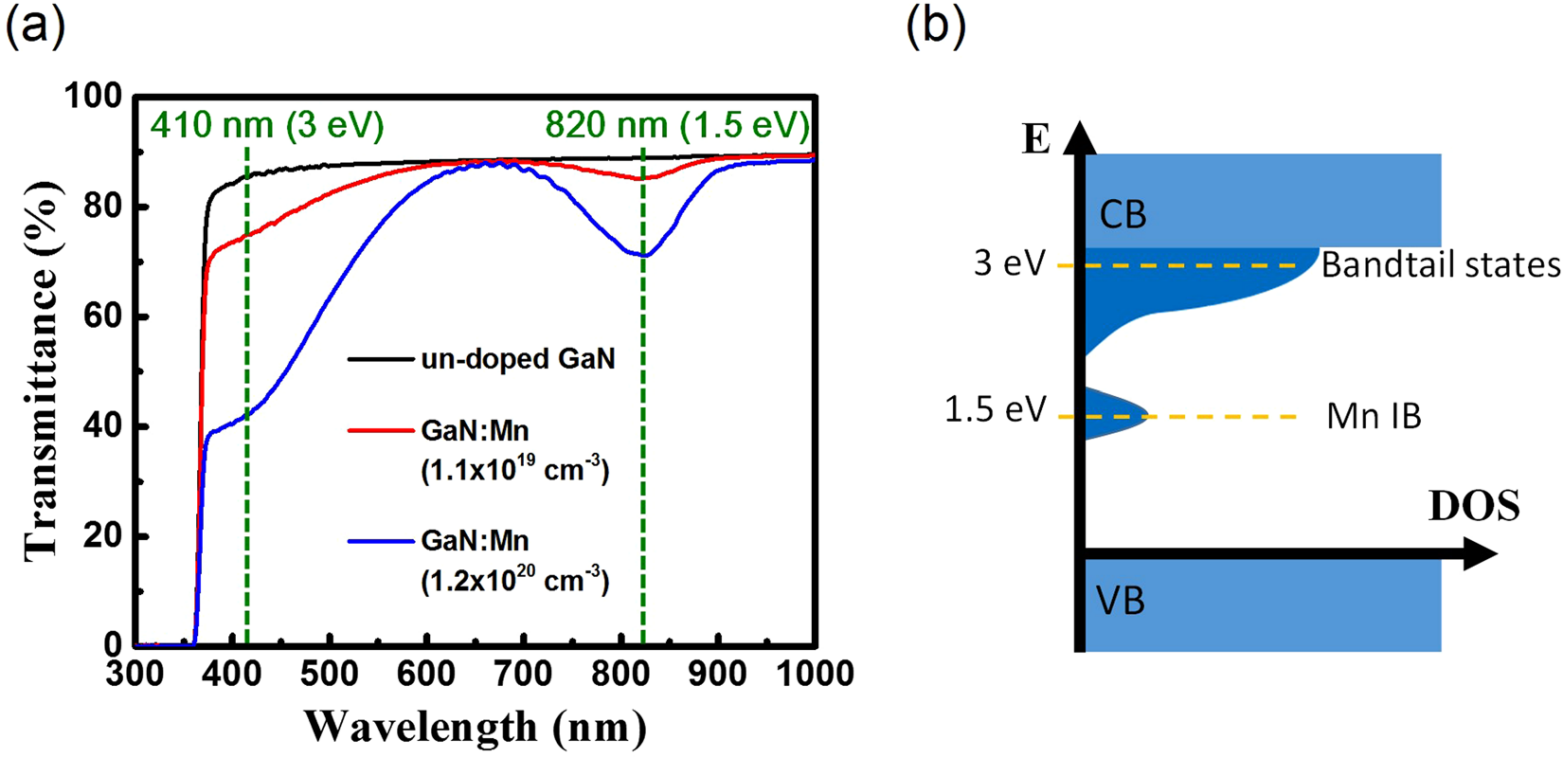
(**a**) The transmission spectra of un-doped GaN and Mn-doped GaN with different doping concentrations. The original spectra obtained by the commercial instrument (Hitachi U-4100) exhibited Fabry-Perot oscillations due to the high-quality films. The transmission spectra here were smoothed out by adjacent averaging to reveal the intrinsic absorption properties. (**b**) The schematic diagram illustrates the density of states in Mn-doped GaN.

**Figure 2:**
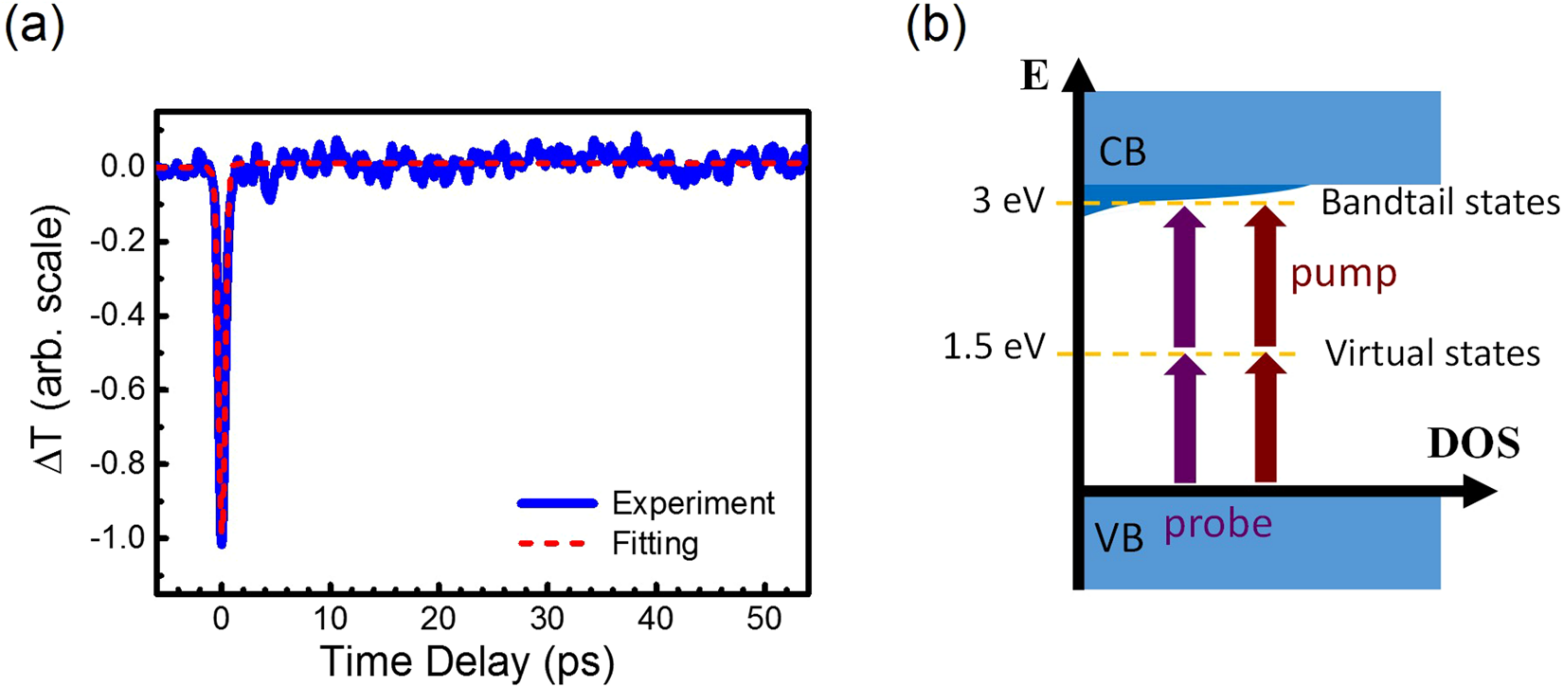
(**a**) The transient transmission changes of the probe pulses through the un-doped GaN. The pump/probe wavelength (photon energy) is 820 nm (1.5 eV). (**b**) The schematic diagram illustrates the density of states in un-doped GaN. Since there is no state at 1.5 eV in un-doped GaN, the transient response in (**a**) is associated with carrier dynamics in the bandtail states monitored by the two-photon transition of the 1.5 eV probe pulses.

Three GaN thin films were studied in this report. One is unintentionally doped GaN (un-doped GaN). The other two were doped with manganese with different concentrations. Figure 1(a) shows the transmission spectra of the three samples. In order to reveal the intrinsic absorption properties, the Fabry-Perot oscillations in the original data were smoothed out. Detailed could be found in the Supplementary Information (SI). The black curve in Fig. 1(a) indicates that the bandgap of GaN is around 3.4 eV (365 nm). It also exhibits little absorption below 3.4 eV, which is attributed to the bandtail states of un-doped GaN. The transmission spectra of Mn-doped GaN with concentrations of 1.1 × 10^19^ cm^−3^ and 1.2 × 10^20^ cm^−3^ are shown in red and blue curves, respectively. They reveal strong dependence on the concentration of Mn. The absorption bands, centered at 820 nm (1.5 eV) and shorter than 600 nm (2.1 eV), both increase absorption with increasing Mn dopants. According to the theoretical calculations^33, 34^, the 3d orbital electrons of Mn atoms (3–5% replacement) additionally contribute the density of states (DOS) in the forbidden band of intrinsic GaN. Part of them are just below the conduction band edge and could extend over 1 eV from the edge^33, 34^. The others are located 1–1.5 eV above the valence band edge^33, 34^. Although the concentrations of Mn in our samples is much lower than 1%, they still contribute significant absorption as shown in the transmission spectra. The experimentally retrieved absorption spectra are shown in the supplementary Fig. S2 of the SI. According to the transmission spectra, the schematic diagrams of DOS such as Fig. 1(b) for Mn-doped GaN and Fig. 2(b) for un-doped GaN are used for assisting interpretation. The Mn-related states, below the conduction bands, are termed as bandtail states. The absorption band, centered at 1.5 eV, is termed as Mn IB.

## Results and Discussions

We first aimed to study the carrier relaxation in Mn IB. The un-doped GaN was treated as the sample for control measurement because Mn IB states in Fig. 2(b) should not exist in un-doped GaN. This is also confirmed by the transmission spectrum in Fig. 1(a). The transient result with pump/probe wavelength of 820 nm (1.5 eV) for un-doped GaN is shown in Fig. 2(a). It appeared a Gaussian-like dip at zero time delay, following slight transmission changes, which was comparable to the noise level of the experimental setup^35^. Because no transition in un-doped GaN is allowed for 1.5 eV photons, we argue that the carrier dynamics in Fig. 2(a) is associated with a small amounts of carriers in the bandtail states. As shown in Fig. 2(b), they could be excited through two-photon absorption (TPA) via a virtual state. The existence of bandtail states, at 3 eV (410 nm) above the valence band edge in un-doped GaN, is also supported by the transmission spectrum in Fig. 1(a).

A response function, composed of functions associated with modeled processes, was used to quantitatively analyze the results. The response function $S(t)$ can be represented as

1 $$S(t)={D}_{0}\delta (t)+{A}_{0}U(t)+[\sum _{i=1}^{N}{A}_{i}\exp (-\frac{t}{{\tau }_{i}})]U(t),$$

where $\delta (t)$ is the delta function representing any instantaneous process such as TPA^31, 36^. $U(t)$ is the step function. ${A}_{0}$ is used for slow responses which exceed the observation windows. ${A}_{i}\exp (-t/{\tau }_{i})$ represents the relaxation process with a characteristic time ${\tau }_{i}$. Because of the finite duration of the optical pulses, the transient transmission changes $\Delta T(t)$ of the experimental data were fitted with the convolution of the response function $S(t)$ and the cross-correlation function $C(t)$ of the optical pump and probe pulses^29, 37^. Here, we let $C(t)=\exp (-{t}^{2}/{\tau }_{d}^{2})$, where ${\tau }_{d}$ characterizes the duration of the cross-correlation. The duration of $C(t)$ varied between 500 fs and 900 fs for our four different experimental setups. The relatively long duration of the optical pulses resulted from the dispersion of the electro-optical modulator (EOM) or acousto-optical modulator (AOM) as described in the Methods section.

Because TPA can be treated as an instantaneous process, the Gaussian-like function at zero time delay in Fig. 2(a) is actually the convolution of ${D}_{0}\delta (t)$ in Eq. (1) and the cross-correlation function of the optical pump and probe pulses $C(t)$ ^31, 36^. Note that the TPA, centered at zero time delay, was simultaneously contributed by each photon from the pump and probe pulses^31^. TPA only occurs when the pump pulse is temporally overlapped with the probe pulse, leading to reduction of the transmitted probe intensity. The red dashed line shows the fitting curve in Fig. 2(a). The parameters of the response function are summarized in Table 1. In addition to ${D}_{0}\delta (t)$ for TPA process, a small value of *A*_0_, corresponding to a slow process within the observation window, was used for fitting. Since the signal is comparable to the noise level, carrier dynamics of un-doped GaN as illustrated in Fig. 2(b) will not be further discussed under this experimental condition.

**Table 1:** The fitting parameters of the response functions in Eq. (1) of each figure. Note that the amplitudes such as *D*_0_, *A*_0_, and *A*_1_ are not comparable between figures.

Figure	*D*_0_	*A*_0_	*A*_1_	τ_1_	*A*_2_	τ_2_	*A*_3_	τ_3_
2 (a)	−1.00	0.01	None	None	None	None	None	None
3 (a)	−0.38	1.09	−2.59	3.9 ps	None	None	None	None
4 (a)	−0.69	0.64	−2.33	2.8 ps	0.79	67.7 ps	None	None
5 (a)	−0.14	2.82	−11.2	4.1 ps	2.15	74.6 ps	None	None
6 (a)	−0.65	2.20	−8.00	3.8 ps	2.69	28 ps	4.30	270 ps
4 (b)	None	None	None	None	0.85	0.18 ns	1.19	1.68 ns

But the scale of amplitudes in each figure keep comparable. The parameters of Fig. 4(b) were obtained without convolution process.

**Figure 3:**
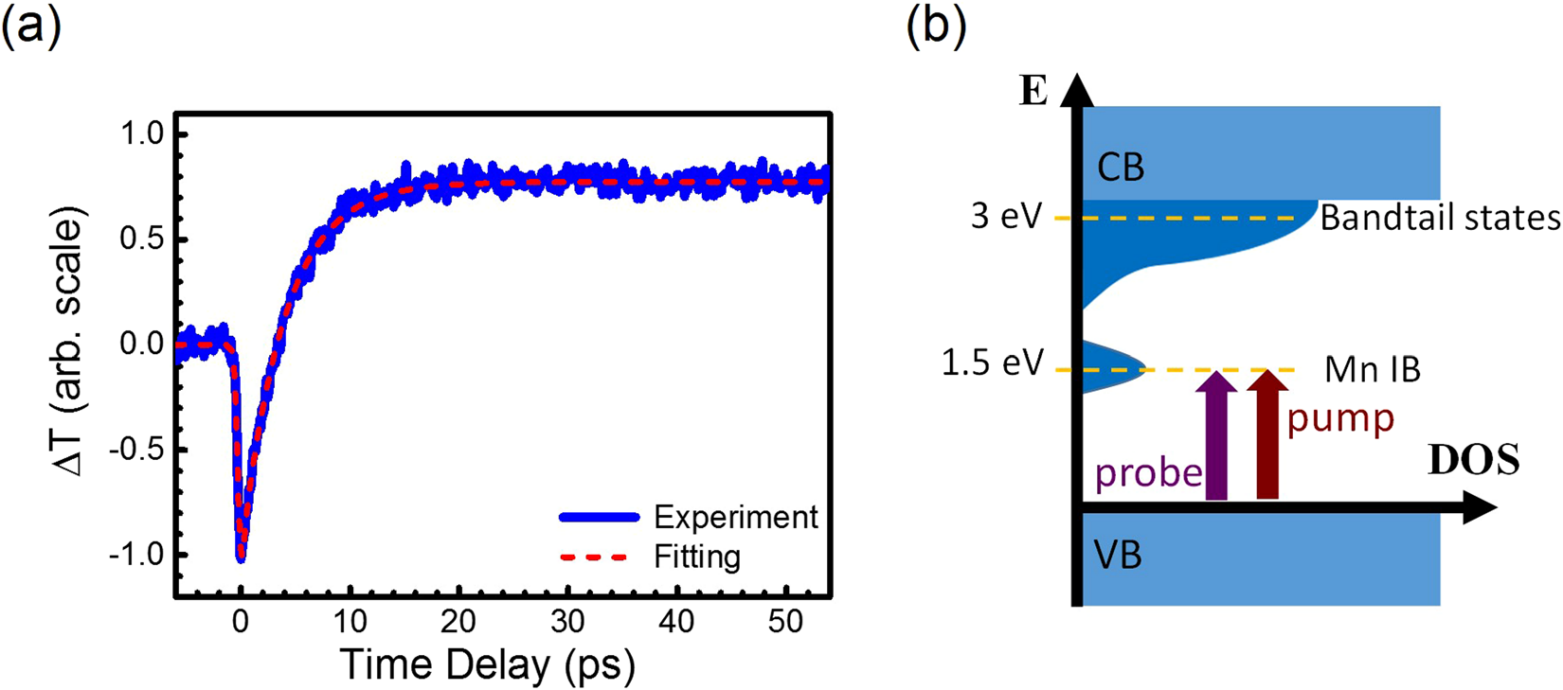
(**a**) The transient transmission changes of the probe pulses through the Mn-doped GaN with concentrations of 1.1 × 10^19^ cm^−3^. The pump/probe wavelength (photon energy) is 820 nm (1.5 eV). (**b**) The schematic diagram illustrates the density of states in Mn-doped GaN. The slow response in (**a**) is associated with carrier dynamics in the Mn IB states monitored by the 1.5 eV probe pulses.

Figure 3(a) shows the transient measurement of Mn-doped GaN with concentrations of 1.1 × 10^19^ cm^−3^. In contrast to the result of un-doped GaN in Fig. 2(a), more significant features appear after TPA at zero time delay. As shown in Table 1, ${D}_{0}\delta (t)$, ${A}_{0}U(t)$ and ${A}_{1}\exp (-t/{\tau }_{1})$ in the response function of Eq. (1) were used for quantitative analysis. Similarly, the delta function ${D}_{0}\delta (t)$ was used to account for the instantaneous process of TPA. The positive step function ${A}_{0}U(t)$ should be attributed to the band-filling effects. After the pump pulses (1.5 eV) excite the electrons to occupy the Mn IB states as shown in Fig. 3(b), the transmission of the probe pulses increase because the partially filled bands reduce the transition probability of the probe pulses. The transient transmission of the probe thus reflects the lifetime of carriers in the Mn IB states. The lifetime of carriers in Mn IB states in Fig. 3(b) is much longer than the observation window of 60 ps. Finally, we suggest that the exponential decay function $\exp (-t/{\tau }_{1})$ with the negative amplitude *A*_1_ is attributed to excited carrier absorption (ECA) or free carrier absorption (FCA). Details will be discussed later before the Conclusions section.

**Figure 4:**
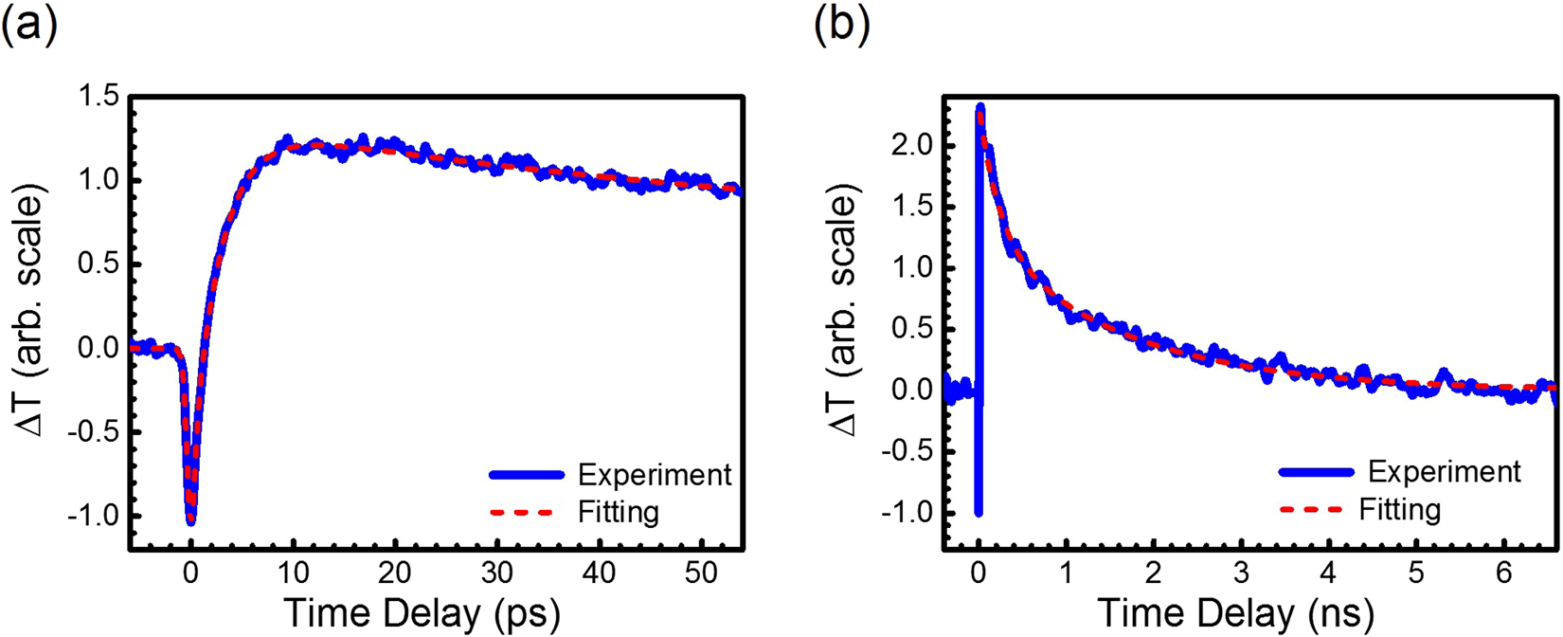
The transient transmission changes of the probe pulses through the Mn-doped GaN with concentrations of 1.2 × 10^20^ cm^−3^. The pump/probe wavelength (photon energy) is 820 nm (1.5 eV). They were monitored by the probe pulses in different experimental setups for (**a**) short and (**b**) long observation windows.

Figure 4(a) shows the transient measurement of Mn-doped GaN with concentration of 1.2 × 10^20^ cm^−3^. The concentration of Mn dopants has an order of magnitude increase, which resulted in good signal to noise ratios. In contrast to the fitting parameters of Fig. 3(a) in Table 1, one more exponential decay function ${A}_{2}\exp (-t/{\tau }_{2})$ was used for fitting. Its physical origin will also be discussed later. Compared with the results of Mn-doped GaN with concentration of 1.1 × 10^19^ cm^−3^ such as supplementary Fig. S3 in the SI, high concentration of Mn dopants led to good transient signals even with 3-fold-lowered pumping power. We thus further used another experimental geometries to investigate the carrier dynamics of Mn-doped GaN with concentration of 1.2 × 10^20^ cm^−3^ to clarify the physical origins in the response functions of Eq. (1) and Table 1.

Figure 4(b) shows the transient response within time window of 7 ns by using an experimental setup with a long linear stage. The transient response recovered into the steady state (or noise level) after 6 ns, qualitatively indicating the lifetime of carriers in Mn IB states. Because of the long observation window, the step function for Fig. 3(a) can be replaced by an exponential decay function with positive amplitude ${A}_{3}\exp (-t/{\tau }_{3})$ for Fig. 4(b) in Table 1. No step function was required for fitting because all responses are back to equilibrium states within the time window. Note that we did not use convolution for fitting because the sampling resolution of time ~10 ps in Fig. 4(b) is much larger than the duration of the cross-correlation $C(t)$. The relaxation on the order of a few ps, as shown in Figs 3(a) and 4(a), thus did not be fitted either. As shown in Fig. 3(b), the lifetime of the excited carriers in the Mn IB states can be monitored, through the aforementioned band-filling effects, by the transmission of the 1.5 eV probe pulses. The time constant τ_3_ = 1.68 ns is attributed to the carrier lifetime in the Mn IB states. Considering that the lifetimes of carriers in other defect-related states of GaN are on the μs timescale^32^, the lifetime of 1.7 ns in Mn IB states is relatively short. The timescale of ns is on the same order as the diffusion time of carriers in GaN^1, 26^. However, the Mn IB states are theoretically contributed from the 3d orbital electrons of Mn atoms^33, 34^. Hopping of electrons between Mn atoms should not occur in our dilutely doped GaN. Electrons in the Mn IB state should be localized, and carrier diffusion was ruled out in our analysis. The dependence of spot size to the relaxation time τ_3_ was also investigated to support this argument.

In Table 1, we argue that τ_2_ = 67.7ps for Fig. 4(a) is attributed to the carrier dynamics within the bandtail states, instead of relevant to the Mn IB states. This is similar to the case of pump excitation in Fig. 2(b) for un-doped GaN. The main difference is that the virtual states are replaced by the real states of Mn IB. It was reported that the real states would greatly enhance the probability of TPA over 1000 times compared with the virtual states^31^. As shown in Fig. 3(b), the 1.5 eV pump pulses could generate significant population of carriers in the bandtail states through TPA via the real state Mn-IB. Compared with un-doped GaN, there are much more bandtail states for pump excitation. On the other hand, the relaxation of carriers in the bandtail states was also probed by the TPA of 1.5 eV pulses via the band-filling effects. Since the generation and detection of carriers in the bandtail states are both through the nonlinear effects, the multiplication of both effects should greatly enhance the nonlinear dependence of Mn concentrations. This should explain that the additional relaxation component with time constant of τ_2_~70 ps appeared in Fig. 4(a) for dopant concentration of 1.2 × 10^20^ cm^−3^, while this component did not apparently appear in Fig. 3(b) for concentration of 1.1 × 10^19^ cm^−3^.

**Figure 5:**
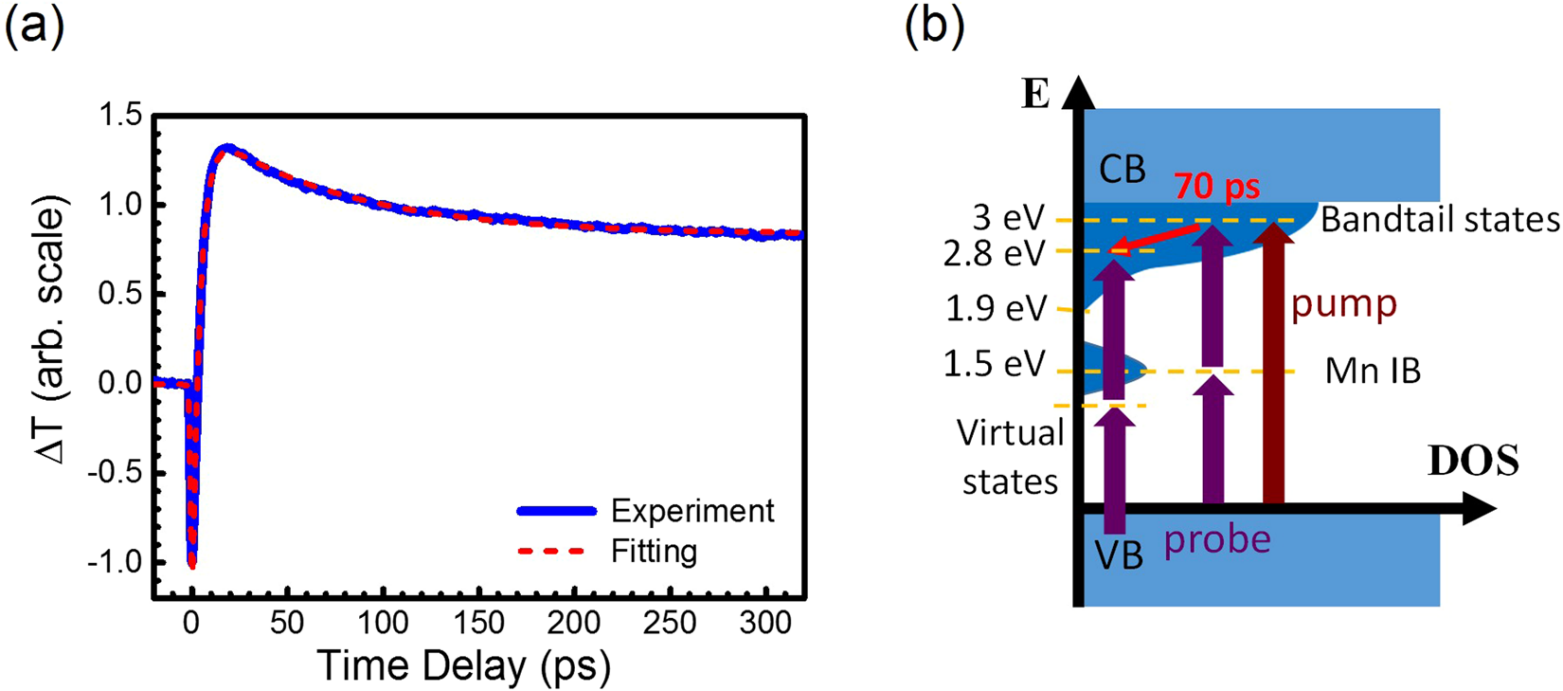
(**a**) The transient transmission changes of the probe pulses through Mn-doped GaN with concentrations of 1.2 × 10^20^ cm^−3^. The pump/probe energy is 3.0/1.5 eV (410/820 nm). (**b**) The schematic diagram illustrates the carrier dynamics in the bandtail states monitored by the two-photon absorption of the 1.5 eV probe pulses. The monitor efficiency is dramatically reduced when the intermediate states become virtual states for two-photon absorption.

For clarification, we used another experimental setup to conduct 410/820 nm pump-probe measurements for the same Mn-doped GaN thin films. As shown in Fig. 5(b), carriers are directly pumped into the bandtail states with single photon absorption of 3.0 eV photons. The carrier dynamics was probed with the same photon energy 1.5 eV in Fig. 4. The transient response is shown in Fig. 5(a), and the parameters for fitting are also summarized in Table 1. Similar to the response function of Fig. 4(a) in Table 1, ${A}_{2}\exp (-t/{\tau }_{2})$ with ${\tau }_{2}=74.6\,{\rm{ps}}$, is attributed to the carrier dynamics within the bandtail states. The time constants τ_2_ of Figs 4(a) and 5(a) are both around 70 ps, indicating that they are from the same physical processes. The carriers could either be excited by the 1.5 eV pump through TPA in Fig. 4(a) or by the 3.0 eV pump through single-photon absorption in Fig. 5(a). But the same subsequent carrier dynamics at 3 eV was monitored by the probe pulses with the same photon energy 1.5 eV, leading to similar τ_2_ for both.

**Figure 6:**
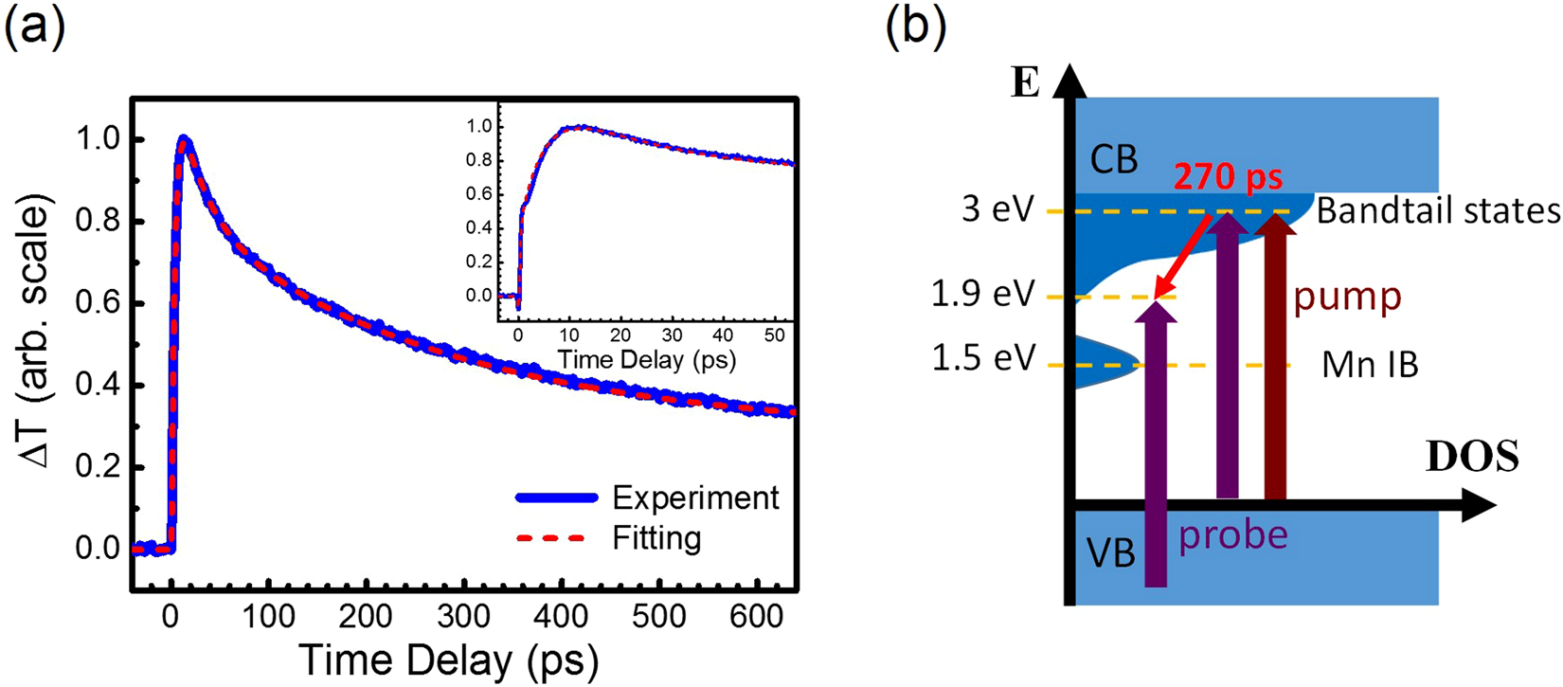
(**a**) The transient transmission changes of the probe pulses through Mn-doped GaN with concentrations of 1.2 × 10^20^ cm^−3^. The pump/probe photon energy is 3 eV (410 nm). (**b**) The schematic diagram illustrates the carrier dynamics in the bandtail states monitored by the 3 eV probe pulses.

Note that τ_2_~70 ps does not reflect the relaxation time of carriers to the edge of the bandtail states. The bandtail states cover the energy range 1.5 eV. As shown in the schematic diagram of Fig. 5(b), it requires energy loss of 1.1 eV for carrier relaxation from 3.0 eV to the edge of the bandtail states at 1.9 eV. However, there is energy range for dramatic enhancement of two-photon transition by the real states. According to the transmission spectra of Mn-doped GaN in Fig. 1(a), the full width at half maximum (FWHM) of Mn IB DOS is ~0.2 eV. The probe mechanism for carriers below 2.8 eV in Fig. 5(b) becomes TPA via the virtual states. Due to the limited bandwidth of the DOS distribution to dramatically enhance TPA of the 1.5 eV probe pulses, τ_2_~70 ps in Figs 4(a) and 5(a) should only reflect the time of carrier relaxation from 3.0 eV to 2.8 eV instead of to the edge of the bandtail states at 1.9 eV. This argument was supported by the results of another experimental setup for 410/410 nm pump-probe geometry in Fig. 6(a).

As shown in Fig. 6(b), the relaxation of carriers from 3.0 eV to 1.9 eV in the bandtail states could be probed by single-photon transition, which was not limited by the bandwidth of the DOS of Mn IB. We found that the whole relaxation in the bandtail states could not be fitted with an exponential decay. Two exponential decay functions with τ_2_ = 28ps and τ_3_ = 270 ps were used to model the relaxation of carriers in the bandtail states. The carrier lifetime in the bandtail states of Mn-doped GaN is on the order of a few hundred ps, which is similar to that of un-doped GaN^28^.

Finally, Table 1 show that the exponential decay function with negative amplitude ${A}_{1}\exp (-t/{\tau }_{1})$, occurred in Mn-doped GaN for Figs 3(a)–6(a). The time constants are 3–4 ps for all the above mentioned figures. The inset of Fig. 6(a) qualitatively highlights ${A}_{1}\exp (-t/{\tau }_{1})$. The negative *A*_1_ led us to attribute this component to excited carrier absorption (ECA) or free carrier absorption (FCA). When carriers are generated in the excited states, they can further transit into another high energy bands by the absorption of the optical probe pulses. Since absorption should occur from occupied low-excited-states to unoccupied high-excited-states, the absorption probability of the probe pulses increases after the pump pulses generate carriers in the low-excited-states, leading to reduced transmission of the probe. The characteristic time τ_1_, on the order of a few picoseconds in Table 1, should also reflect the lifetime of the carriers in the low-excited-states.

For 820 nm degenerate pump-probe measurements in Fig. 3(a and b), it might be straightforward but incorrect to attribute the ECA to the transition from the Mn IB states to the bandtail states. Because the ECA from the Mn IB states and the band-filling effects both reflect the carrier lifetime in the Mn IB, they should be modeled with the same exponential functions. In other words, they are not distinguishable in only one ${A}_{i}\exp (-t/{\tau }_{i})$. For 820 nm degenerate pump-probe measurements in Figs 3(a) and 4, the lifetimes of carriers in the Mn IB states were dominated by the band-filling effects because under our experimental conditions, only 10^–16^ cm^−3^ carriers at most were generated in the Mn-IB states of Mn-GaN. Transition of the probe pulse from the valence band to the Mn-IB should overwhelm the effects of ECA from the Mn-IB states.

${A}_{1}\exp (-t/{\tau }_{1})$ for Figs 3(a)–6(a) should not associate with the photoexcited carriers in the bandtail states, either. According to our measurements with different pump/probe geometries, the carrier relaxation time in the bandtail states is on the order of a few hundred ps. These results led us to suggest that the ECA of Mn-doped GaN should attribute to the free carriers in the conduction bands, that is, FCA. The lifetimes of 3-4 ps also agree with that of FCA in semiconductors in the literature^38^. For pump energy of 1.5 eV, free carriers could be generated via three-photon process. Although the transition rate of three-photon process is much lower than that of TPA and single-photon absorption, the transition probability would be dramatically enhanced due to the real states at 3 eV and 1.5 eV. We thus suggest ${A}_{1}\exp (-t/{\tau }_{1})$ for Figs 3(a)–6(a) are associated with the free carriers in the conduction bands through multi-photon processes of the 1.5 eV and 3 eV pulses.

## Conclusions

We report the carrier dynamics of Mn-doped GaN with doping concentration of 10^19^–10^20^ cm^−3^. According to the transmission spectra, Mn dopants induce intermediate bands at 1.5 eV above the valence band edge. It also extends the energy range of the bandtail states below the conduction band. The excess states enable Mn-doped GaN absorbing visible light and increase the conversion efficiency of GaN-based solar cells. The spectra of absorption coefficients were obtained in supplementary Fig. S2 of the SI, which would be helpful to design the thickness of Mn-GaN thin film for photovoltaics. For example, the absorption length of Mn-doped GaN (10^20^ cm^−3^), at the center of Mn IB, was measured as ~5 μm. A thickness of Mn-GaN such as over 10 μm should be suggested for optimization of photon collection.

The lifetime of carriers in the Mn-related IB was obtained to be 1.7 ns. The carrier relaxation time within the bandtail states was on the order of a few hundred ps. It was reported that the carrier relaxation times of IB states of InAs/GaAs quantum dot systems are 0.3–0.9 ns^39^, which was similar to the relaxation time of carriers in the Mn-induced states of GaN. Carrier relaxation times or lifetimes are one of parameters to model the dynamics of intermediate-band solar cells^13^. Understanding carrier dynamics in Mn-doped GaN should lead to some hints for improvement of the conversion efficiency through photo-induced carriers in the intermediate bands. For example, the experimentally obtained relaxation times in the Mn-induced states could be considered in the model for simulation and optimization of the photovoltaic devices.

## Methods

### Sample Preparation

The 30 nm-thick GaN nucleation layers were firstly grown on the double polished c-plane sapphire substrates at low temperature (550 °C) with metal-organic chemical vapor deposition (MOCVD). On the top of the GaN nucleation layer, un-doped GaN thin film was grown at 1000 °C and followed by epitaxy of GaN with different flow rates of Mn source [(MeCp)_2_Mn] for the three samples. The gas flow rates of Mn were maintained at 0, 50, and 500 sccm during growth process, respectively. The epitaxial durations of the top layers were kept the same. According to previous measurements using secondary ion mass spectrometry (SIMS) with the same growth conditions, Mn-doped GaN with flow rates of 50 and 500 sccm should exhibit Mn concentrations on the order of 1.1 × 10^19^ cm^−3^ and 1.2 × 10^20^ cm^−3^, respectively. Mn atoms should be dilutely distributed according to the images of transmission electron microscope (TEM) with magnification of 0.6 million. Detailed information about the samples can be found in the SI.

### Sample Information

The un-doped GaN layer exhibited n-type conduction, and the typical electron concentration determined by Hall measurement was approximately 5 × 10^16^ cm^−3^. However, the resistivity of Mn-doped GaN thin films exceeded ${10}^{11}\,\Omega \cdot {\rm{cm}}$, which is the instrument limit. It should be noted that the solar cell based on Mn-doped GaN should work under light exposure to consistently excite electrons from the Mn-related states to the conduction bands. Because the Mn-related states would trap the free carriers, the ultrahigh resistivity of Mn-doped GaN is owing to the lateral electrical measurement with large scales for Hall-effect measurements and the absence of significant intensity of light exposure during the electrical measurements.

The thickness of un-doped GaN was estimated as 6.8 μm, according to the Fabry-Perot oscillations of the transmission spectra in supplementary Fig. S1 of the SI. The thicknesses of Mn-doped GaN thin films, on the 4.9 μm-thick un-doped GaN, were estimated as 1.8 μm and 1.1 μm for Mn concentrations of 1.1 × 10^19^ cm^−3^ and 1.2 × 10^20^ cm^−3^, respectively. Moreover, the absorption coefficients of Mn-doped GaN were experimentally obtained as shown in supplementary Fig. S2 of the SI. The absorption coefficients at 820 nm, corresponding to the Mn-related IBs, were estimated to be 0.023 μm^−1^ and 0.200 μm^−1^ for low and high Mn concentrations, respectively. Regarding to the excitation wavelength at 410 nm, the absorption coefficients were 0.076 μm^−1^ and 0.654 μm^−1^ for low and high Mn concentrations, respectively.

### Experimental Setup

Optical pulses, centered at 820 nm, were used from a Ti:sapphire laser with repetition rate of 80 MHz. Typical non-collinear pump-probe measurements were conducted with three different geometries 820/820 nm, 410/820 nm, and 410/410 nm. Optical pulses, centered at 410 nm, were generated through a BBO nonlinear crystal. The polarization of the pump beam was orthogonal to that of the probe beam. A pinhole was used to secure the spatial overlap of the focused pump and probe spots, and to obtain the spot sizes. The optical intensity distribution was assumed to follow $\exp (-2{r}^{2}/{w}^{2})$, and 2*w* was defined as the spot size here. For 820 nm degenerate pump-probe measurements, the spot sizes of the pump and probe focused on the sample were measured to be ~11 μm and ~19 μm by a 10 μm pinhole. A polarizer was placed in front of the photodetector to eliminate the pump leakage light. The pump beam was modulated at 10 MHz with an EOM or 100 kHz with an AOM. A lock-in amplifier was used to record the transmission variation of the probe pulse as a function of time delay between the pump and probe pulses. For 410 nm pump geometries, the pump beams were modulated at 1 MHz with an AOM. The optical fluences of the probe beams were much lower than that of the pump beams for all the measurements.

### Experimental Conditions

For the traces in Fig. 4, the 820 nm pump fluence was 46 μJ/cm^2^. According to the absorption coefficient obtained in the SI, the concentration of excited carriers in Mn IB states was estimated to be 3 × 10^15^ cm^−3^. The optical fluences for Fig. 3(a) were 2–4 times higher. But the absorption was much weaker, leading to low signals of transmission changes in Fig. 3(a). The fluences were within weak perturbation regime and linear range, which was confirmed by the power-dependent measurements as shown in supplementary Fig. S2 in the SI. For 410 nm excitation in Figs 5 (a) and 6(a), the pump fluence was 71 μJ/cm^2^, resulting in excited carrier population of 7 × 10^15^ cm^−3^ in the bandtail states.

## Electronic supplementary material

Supplementary Information
