# Supplementary material for: Carrier dynamics of Mn-induced states in GaN thin films
Source: Sci Rep. 2017 Jul 19;7:5788. doi: 10.1038/s41598-017-06316-7 (PMC5517569; doi:10.1038/s41598-017-06316-7)
Supplement: Supplementary file 1 — Supplementary Information [file 41598_2017_6316_MOESM1_ESM.pdf]

Supplementary Information to

Carrier dynamics of Mn-induced states in GaN thin films

Yu-Ting Chen,[1] Chi-Yuan Yang,[1,2] Po-Cheng Chen,[3] Jinn-Kong Sheu,[3] and Kung-Hsuan Lin[1,4*]

[1]Institute of Physics, Academia Sinica, Taipei 11529, Taiwan

[2]Department of Physics, National Taiwan University, Taipei 10617, Taiwan

[3]Department of Photonics, National Cheng Kung University, Tainan 70101, Taiwan

[4]Institute of Photonics Technologies, National Tsing Hua University, Hsinchu 30013, Taiwan

[*]E-mail Address: kunghsuan@gmail.com

Measurements of Mn concentrations

In order to measure the doping concentrations, the Mn-doped GaN thin films were grown on the un-doped GaN with metal-organic chemical vapor deposition (MOCVD). During the growth process, flow rates 50, 100, 300, and 500 sccm of Mn source [$(MeCp)_2Mn$] were used. Except the flow rates, all the experimental conditions were kept the same. These samples were sent to Evans Analytical Group (Sunnyvale, CA, USA) for secondary ion mass spectroscopy. To perform the calibration of Mn concentration in the Mn-doped GaN, the reference samples were prepared by Mn ion implantation with a given dosage into GaN. Table S1 summarizes the reported values without error range from Evans Analytical Group. In this work, two samples with flow rates of 50 and 500 sccm were prepared under the sample experimental conditions, the corresponding concentrations of Mn should be 1.1×10^{19} cm^{-3} and 1.2×10^{20} cm^{-3}, respectively.

CP2Mn flow	CP2Mn mole flow rate	Concentration of Mn
50 sccm	0.185 μmole/min.	$1.1x10^{19}/cm^3$
100 sccm	0.37 μmole/min.	$1.2x10^{19}/cm^3$
300 sccm	1.11 μmole/min.	$6.5x10^{19}/cm^3$
500 sccm	1.85 μmole/min.	$1.2x10^{20}/cm^3$

Table S1. The doping concentration of Mn in GaN with different flow rates of $(MeCp)_2Mn$.

Thickness of Mn-doped GaN thin films

The Fabry-Perot oscillations can be used to characterize the thickness of the GaN films. In addition to Fig. 1 (a), we performed another measurements of transmission spectra with the instrument (MFS-630, Hong-Ming Technology Co., Ltd) specifically for the purpose of thickness analysis. White light from a low-power halogen lamp was normally incident to the samples. The diameter of the illuminated area was within 1 mm. The transmitted light was collected with an integrating sphere. Fig. S1 shows the transmission spectra of Mn-doped GaN with different concentrations. In contrast to the spectra in Fig. 1 (a), the effective wavelength range of Fig. S1 is narrow because of the restrictions of the light sources and detectors. The absorption of the three samples is lowest at ~ 660 nm. It should be reasonable to assume that the difference of refractive indexes between un-doped GaN and Mn-doped GaN is ignorable around 660 nm.

The period of the optical frequency $\Delta\nu = c/(2nh)$, where c is the speed of light in vacuum. n and h are the refractive index and the thickness of GaN, respectively. We measured $\Delta\nu$ for two oscillating cycles around 660 nm in Fig. S1 and treated the refractive index as a constant 2.38 [S1]. $\Delta\nu$ of 9.3 THz, 9.4 THz, and

10.5 THz correspond to GaN total thickness of 6.8 μm, 6.7 μm, and 6.0 μm for Mn concentrations of 0, 1.1×10^{19} cm^{-3} and 1.2×10^{20} cm^{-3}, respectively. The thicknesses of Mn-doped GaN thin films, on the 4.9 μm-thick un-doped GaN, were thus estimated as 1.8 μm and 1.1 μm for low and high Mn concentrations, respectively. The experimentally obtained values of thickness, at different measured spots within 1 cm by 1 cm, varied less 5 %. It could result from the accuracy of determining the oscillation periods in the absorption spectra. However, it does not affect the correctness of investigating carrier dynamics even though all the errors are attributed to the thickness variation of Mn-doped GaN thin films.

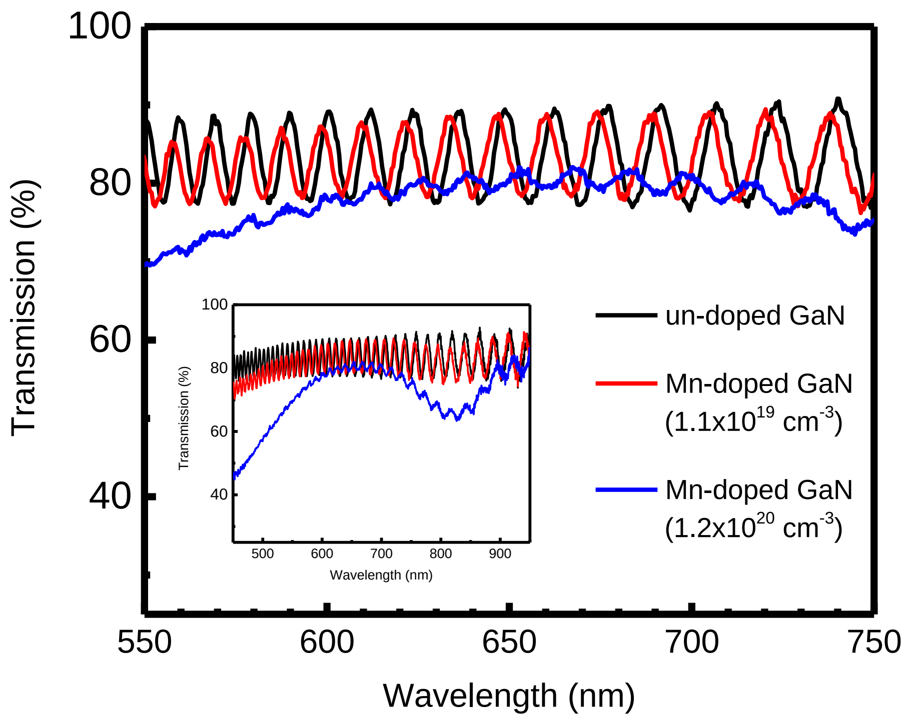

Figure S1. The transmission spectra of the samples. The absorption is lowest around 660 nm, which is suitable for calculations of film thickness from the Fabry-Perot oscillations. The inset shows the effective range of the instrument with a low-power light source of halogen lamp.

Absorption coefficients of Mn-doped GaN thin films

The absorption coefficients could be obtained from the transmission spectra if the thickness of absorbing materials is determined. Since the absorption of un-doped GaN for the wavelength range longer than 400 nm could be neglected, it can be treated as the reference sample to calibrate the reflection loss at the interfaces. The spectra in Fig. 1 (a) were normalized to the spectrum of un-doped GaN, and following the loss was attributed to the absorption. According to Beer's law, the transmission rate is $\exp(-\alpha L)$, where α and L are absorption coefficient and absorption length. The absorption spectra of Mn-doped GaN thin films were obtained as shown in Fig. S2.

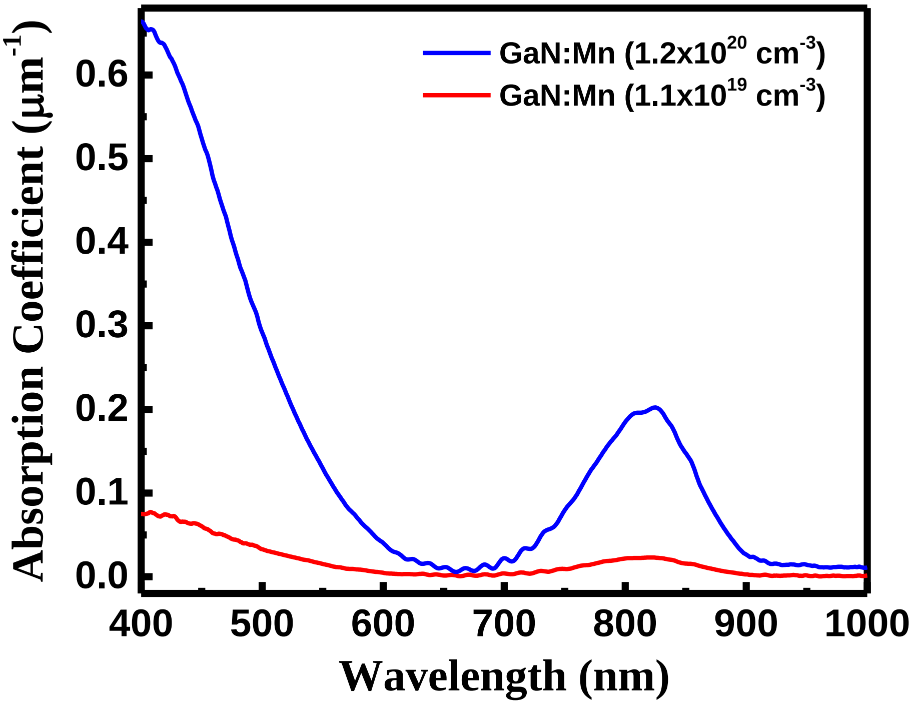

Figure S2. Absorption coefficients of Mn-doped GaN as a function of wavelength, calculated from the experimental data in Fig. 1 (a).

Power-dependence of transient transmission through Mn-doped GaN

We have conducted pump-power-dependent, 820 nm (1.5 eV) degenerate pump-probe experiments. Fig. S3 show the results of Mn-doped GaN with doping concentrations of 1.1×10^{19} cm^{-3} and 1.2×10^{20} cm^{-3}, respectively. Both of them reveal linearity. They confirm that the pump fluences we used were within weak perturbation regime and linear range. High concentration of Mn dopants gave rise to high density of states of Mn-IB at 1.5 eV above the valence band edge, leading to good transient signals. Although the power for Fig. S3 (b) is lower compared with Fig. S3 (a), better signals were still achieved.

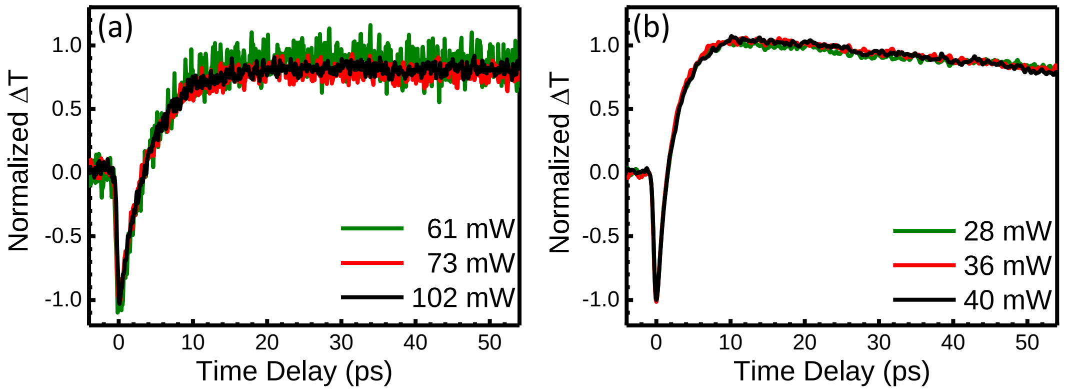

Figure S3. The power-dependence of transient transmission through the Mn-doped GaN with doping concentrations (a) 1.1×10^{19} cm^{-3} and (b) 1.2×10^{20} cm^{-3}. The traces are normalized for comparison.

References

[S1] https://refractiveindex.info; A. S. Barker Jr. and M. Ilegems. Infrared Lattice Vibrations and Free-Electron Dispersion in GaN. Phys. Rev. B **7**, 743-750 (1973).
